# Mitophagy as a pivotal axis in non-alcoholic fatty liver disease: From pathogenic mechanisms to therapeutic strategies (Review)

**DOI:** 10.3892/mmr.2025.13664

**Published:** 2025-08-27

**Authors:** Yushu Huang, Xueqing Xia, Jingyang Xu, Zihan Wang, Yanting You, Qingfeng Du

**Affiliations:** 1 School of Traditional Chinese Medicine, Southern Medical University, Guangzhou, Guangdong 510515, P.R. China; 2 Southern Medical University Hospital of Integrated Traditional Chinese and Western Medicine, Southern Medical University, Guangzhou, Guangdong 510315, P.R. China; 3 Guangdong Provincial Key Laboratory of Chinese Medicine Pharmaceutics, Southern Medical University, Guangzhou, Guangdong 510515, P.R. China; 4 Guangdong Basic Research Center of Excellence for Integrated Traditional and Western Medicine for Qingzhi Diseases, Guangzhou, Guangdong 510315, P.R. China

**Keywords:** non-alcoholic fatty liver disease, mitophagy, mechanisms, therapies

## Abstract

Non-alcoholic fatty liver disease (NAFLD), characterized by excessive lipid accumulation in hepatocytes, has emerged as the leading cause of chronic liver disorders globally. As the central metabolic organ, the liver critically depends on mitochondrial integrity. Mitophagy, a selective form of autophagy, plays a pivotal role in sustaining mitochondrial homeostasis by eliminating dysfunctional mitochondria. Dysregulated mitophagy contributes to the progression of NAFLD, while its restoration mitigates disease severity. The present review outlines the tripartite axis of mitophagy, namely, the PTEN-induced putative kinase 1/Parkin, PI3K/AKT/mTOR and AMP-activated protein kinase pathways, in NAFLD pathogenesis across the various stages of disease development, including steatosis, nonalcoholic steatohepatitis and fibrosis, and explores their therapeutic potential. Additionally, emerging regulators, including FUN14 domain-containing protein 1, prohibitin 2, ceramide signaling and non-coding RNAs, which fine-tune mitophagy in NAFLD are highlighted. By integrating evidence from pharmacological and natural agents, including traditional Chinese medicines, mitophagy-centric strategies to promote hepatic lipid metabolism, mitigate disease progression and inform novel NAFLD therapeutics are discussed. This exploration of the mechanisms that govern mitochondrial-autophagic crosstalk not only advances mechanistic insights but also opens new avenues for precision medicine in the treatment of metabolic liver diseases.

## 1. Introduction

Non-alcoholic fatty liver disease (NAFLD) is a metabolic syndrome that is rapidly emerging as a worldwide health concern. Non-alcoholic steatohepatitis (NASH), which is a more severe form of NAFLD, tends to progressively worsen and ultimately result in cirrhosis and hepatocellular carcinoma (HCC) (1). The nomenclature of NAFLD has undergone two major revisions in recent years. First, in 2020, an international expert panel recommended the reclassification of NAFLD as metabolic-associated fatty liver disease, emphasizing that the disease pathogenesis is inherently associated with metabolic abnormalities (2). In 2023, it was proposed by the American Association for the Study of Liver Diseases and the European Association for the Study of the Liver in collaboration with the Asociación Latinoamericana para el Estudio del Hígado that the condition should be renamed as metabolic dysfunction-associated fatty liver disease, with the introduction of the term dysfunction to emphasize the role of metabolic dysregulation in the pathogenesis of the disease (3). The present review systematically discusses classic research on NAFLD, using the term NAFLD to maintain consistency with the historical literature.

As economies have developed, dietary patterns have changed, contributing to a rise in the global prevalance of NAFLD, which was ~30% between 2009 and 2019 and has since continued to increase (4). The rate of increase in NAFLD prevalence in China is particularly notable, being more than twice as rapid as that in Western countries. NAFLD is the leading cause of chronic liver diseases, accounting for 38% of cases globally and 30% in China (5,6). However, due to the complex pathophysiology of NAFLD, no specialized medications are available. Current therapeutic treatments primarily target underlying causes, key pathophysiological mechanisms and associated metabolic abnormalities (7). Therefore, the development of novel therapeutic approaches is crucial to addressing the growing burden of this disease.

Gluconeogenesis, glycogen storage and the metabolism and detoxification of drugs and other exogenous substances are among the numerous vital metabolic processes carried out by the liver (8). Mitochondria play a crucial role in the regulation of lipid utilization, cell division processes and redox equilibrium in hepatic cells (9). Mitochondrial dysfunction has been implicated in acute and chronic liver diseases. Furthermore, numerous studies have shown that the normal functions and disease-associated processes of the liver depend heavily on mitophagy, a type of autophagy that is responsible for the removal of damaged or excessive mitochondria (9). Given that NAFLD may be impacted by mitophagy, the present study discusses the mechanisms of NAFLD from the perspective of mitophagy. By focusing on mitophagy as a potential therapeutic target, the present study aims to provide insights that may inspire future research to reduce the incidence and advancement of NAFLD.

## 2. NAFLD

NAFLD is frequently associated with metabolic disorders including obesity, diabetes, dyslipidemia and hypertension, and is characterized by the overproduction of fatty acids by hepatocytes (7). The ‘two-hit’ hypothesis was originally accepted to explain the pathogenesis of NAFLD, because obesity, diabetes and other risk factors cause insulin resistance (IR), therefore, it is believed that obesity and IR are the main causes of the initial attack. However, due to the complexity of NAFLD, the ‘two-hit’ model is now considered overly simplistic as it cannot adequately explain the development of NAFLD. Consequently, the ‘multiple-hit’ hypothesis was proposed. This suggests that in genetically predisposed individuals, NAFLD develops due to the combined effects of multiple insults, including insulin resistance, adipose-derived hormones, dietary factors (10) and chronic low-grade inflammation (11).

Notably, the spleen, as a primary immune organ, plays a major role in mediating the inflammatory response, which contributes to the induction and worsening of NAFLD (12). In addition, gut dysbiosis, an imbalance in the intestinal microbiota, has been implicated in NAFLD development and progression. Alterations in the gut microbial composition and metabolites are considered to exacerbate NAFLD via mechanisms such as increased intestinal permeability, disrupted food metabolism, intestinal motility disorders and intestinal inflammation (13).

The current treatment methods for NAFLD, include lifestyle modifications, exercise and dietary adaptations, bariatric surgery, and medical therapies such as vitamin E and the proliferation activated receptor gamma (PPAR-γ) ligand pioglitazone (14–16), which are applied to minimize the impact of the disease and slow its course.

## 3. Autophagy

Autophagy is a cellular process in the cytoplasm including dysfunctional organelles, misfolded proteins or invading pathogens are enclosed by a membrane structure called an autophagosome. Autophagosomes then fuse with lysosomes to generate autolysosomes, in which the sequestered contents are broken down, thereby helping to maintain cellular homeostasis and facilitating organelle renewal (17). This degradation process prevents the buildup of noxious substances that could trigger cell death (18). There are multiple types of autophagy. Autophagy can be categorized into three primary types based on the mechanism by which cargo is delivered to lysosomes: Macroautophagy, microautophagy and chaperone-mediated autophagy (19). Alternatively, autophagy can be categorized into non-selective and selective types, based on whether specific recognition mechanisms are involved in the targeting of the cargo.

### Non-selective autophagy

Non-selective autophagy involves the indiscriminate phagocytosis of cell membrane components (20). It functions in a nondiscriminatory manner, without specific targeting. For example, under conditions of starvation or malnutrition, cytoplasmic materials are randomly sequestered for degradation. This process enables the recycling and redistribution of cellular resources such as proteins, carbohydrates and lipids, thereby providing basic maintenance and compensatory mechanisms to support cellular function (21).

### Selective autophagy

Selective autophagy involves the lysosomal breakdown of specific intracellular substances that have been sequestered into lysosomes, late endosomes or autophagosomes. It targets specific substances for degradation, including proteins, organelles and pathogens, by processes which include mitophagy, lysosomal autophagy, proteaphagy and lipophagy. Selective autophagy specifically removes misfolded protein aggregates, damaged or redundant organelles, endoplasmic reticulum (ER), lipid droplets (LDs), and invading bacteria and viruses. Current research indicates that selective autophagy is highly important in the maintenance of liver homeostasis (9).

### Proteophagy

In eukaryotic cells, the autophagy-lysosome, caspase and ubiquitin-proteasome systems are the three traditional pathways that mediate protein degradation (22). The selective degradation of proteins, which are essential for numerous cellular functions, is achieved by proteophagy, an autophagy-lysosome pathway-based mechanism for protein degradation. Notably, proteasomes may also act as autophagy platforms by linking to autophagosomes via p62/SQSTM1, helping recruit and deliver ubiquitinated proteins for autophagic breakdown. When overloaded, entire proteasome complexes can themselves be degraded through proteaphagy, providing a dynamic switch between proteasomal and lysosomal degradation pathways to maintain cellular protein balance (23).

### Lipophagy

The liver, which is the primary location of lipid metabolism, relies on a number of molecular mechanisms to maintain lipid homeostasis, including regulation of fatty acid transport via fatty acid transport protein 1 and fatty acid binding protein, fatty acid β-oxidation mediated by carnitine palmitoyltransferase-1 and lipid metabolism modulation through lipophagy (24,25). Lipid metabolism is associated with lipophagy, since nutritional deficiency triggers the mobilization of free fatty acids (FFAs) to provide fuel under stress conditions, such as starvation and hypoxia (26), and this is promoted by lipophagy, a lysosomal-mediated process in which LDs are delivered to lysosomes for degradation (27–30). Several studies have highlighted the importance of lipophagy in the regulation of cell homeostasis and maintenance of lipid metabolism (25).

### Reticulophagy

The ER is a multipurpose organelle that participates in various biological functions, including protein and lipid production as well as the regulation of cell death. Reticulophagy is a selective mechanism that removes damaged ER components via autophagy-mediated lysosomal breakdown. Due to the dynamic nature of the ER membrane, reticulophagy is important for maintaining the size of the ER and altering its morphology. It mainly approves selective fragmentation into small membrane fragments via ER-phagy receptors, ER stress or the unfolded protein response, so that to maintain ER homeostasis under both stress and normal conditions (31,32). In addition, the selective removal of ER subdomains by reticulopathy helps to maintain ER homeostasis and eliminate dysfunctional ER segments (33). Reticulophagy is a crucial process for quality control of the ER (22).

### Mitophagy

Mitochondria are the main energy-producing organelles in cells; they are present in most eukaryotic cells and serve as the principal site of aerobic respiration. These dynamic organelles have essential functions in various cellular functions and are fundamental to cell metabolism and survival (34). However, mitochondria are vulnerable to damage, which may disrupt cellular homeostasis and lead to mitochondrial diseases. These alterations are closely associated with cancer and metabolic disorders (35,36). Therefore, the prompt elimination of damaged mitochondria is essential, and this occurs via the mitophagy process, a specialized autophagic mechanism that selectively removes surplus and damaged mitochondria to maintain mitochondrial integrity (37). When injured by exposure to external stimuli such as reactive oxygen species (ROS), dietary deficiencies or cell ageing, mitochondria become depolarized and lose membrane potential. At this stage, autophagosomes enclose the mitochondria to create mitophagosomes, which subsequently fuse with lysosomes. The lysosomes break down the mitochondrial components, using lysosomal or vacuolar acid hydrolases to degrade the damaged mitochondria. Mitophagy plays an essential role in maintaining the homeostasis of cellular mitochondria and is vital for overall cellular health (38). Currently, several common signaling pathways are known to regulate mitophagy, including the PTEN-induced putative kinase 1 (PINK1)/Parkin-mediated pathway, the AMP-activated protein kinase (AMPK)-mediated pathway, the PI3K/AKT/mTOR signaling pathway and the BCL2 interacting protein 3 like-mediated pathway (39,40).

## 4. Autophagy and NAFLD

By helping to establish energy balance and preserve the quality of the cytoplasm, autophagy is vital for maintaining liver homeostasis (41). The liver is highly dependent on autophagy due to its role in the mediation of hepatic lipid metabolism. The importance of autophagy and its intimate association with NAFLD is becoming increasingly evident (42). Numerous proteins, including microtubule-associated protein 3 (LC3) and sequestosome 1 (p62), have been implicated in autophagy. For example, reductions in p62 and LC3-II levels have been observed in patients with steatosis and NASH, indicating that autophagic activity is decreased under these conditions (43,44). In addition, in NAFLD ApoE^−/−^ mice, a high-fat diet (HFD) was shown to reduce the expression of the autophagy-related proteins p62 and Beclin 1 (45). Furthermore, ring finger protein 31, a key factor in the initiation of mitophagy and regulation of mitochondrial balance, has been found to reduce steatosis in lipotoxic hepatocytes (46).

### Lipophagy and NAFLD

The dysregulation of lipophagy has been implicated in a number of the pathogenic features of liver disease. In hepatocytes, impaired lipophagy leads to the abnormal buildup of LDs, a defining trait of liver conditions such as NAFLD and NASH (47). The excessive accumulation of LDs within hepatocytes is one of the most well-established characteristics of NAFLD development (48). Lipophagy, a type of autophagy that is specifically implicated in LD degradation, contributes to lipid clearance and has been identified as a pathway contributing to NAFLD development (49). This process involves the autophagic catabolism of LDs through ‘acid’ lipolysis mediated by lysosomal acid lipase in acidic lipolysosomes, serving as an alternative to cytosolic neutral lipolysis pathways for hepatic LD degradation (50,51). Liver lipophagy has been shown to ameliorate NASH through extracellular lipid secretion, activated lipophagy triggers lysosomal deacidification, stimulating transient receptor potential mucolipin 1-mediated Ca^2+^ release and synaptotagmin 7-dependent lysosomal exocytosis, with lipid droplets moving near the cell membrane and increased cell surface LAMP1, thereby excreting lipids to reduce intracellular harmful nonesterified fatty acids (52).

### Mitophagy and NAFLD

Individuals with fatty liver disease commonly exhibit oxidative stress and mitochondrial dysfunction in their liver tissue (10,53). Persistent mitochondrial impairment in NAFLD contributes to a harmful disruption of metabolic balance and also promotes increased ROS generation, lipid peroxidation, cytokine release and cellular death. Patients with NAFLD exhibit increased mitochondrial dysfunction, and those with NASH exhibit enlarged and swollen hepatocellular mitochondria along with structural defects such as cristae deficiency (9,54,55). NAFLD represents a disease spectrum, progressing from non-alcoholic fatty liver (NAFL) with only simple hepatic steatosis to NASH and eventually to liver fibrosis. Notably, the expression levels of markers associated with mitophagy, such as BCL2 interacting protein 3, autophagy related protein 5 and Beclin1, are significantly reduced in NAFL and NASH (56). In addition, the impairment of mitophagy has been observed in animal liver fibrosis models induced by CCl_4_ or common bile duct ligation (57). The present review aims to elaborate on the involvement of mitophagy-related signaling pathways in NAFLD progression in the following sections.

### Mitophagy driven by the PINK1/Parkin pathway and its role in NAFLD

Parkin and PINK1 cooperate with each other and have crucial functions in mitophagy (58). PINK1 is predominantly localized to mitochondria where it plays a vital role in the detection of mitochondrial depolarization, thereby acting as a critical regulator of mitophagy. Parkin, encoded by the PARK2 gene, is an E3 ubiquitin ligase that is also essential to mitophagy. When recruited by PINK1, Parkin labels the cell membrane by the addition of ubiquitin, thereby marking the organelle for degradation by the proteasome or lysosome. Deoxycholic acid, a secondary bile acid formed by the removal of a hydroxyl group from bile acid, has been shown to inhibit PINK1-mediated mitophagy in FFA-stimulated fatty liver cells. This inhibition leads to the activation of the Nod-like receptor family pyrin domain containing 3 (NLRP3) inflammasome and the induction of hepatocyte apoptosis (59).

**Figure 1.**
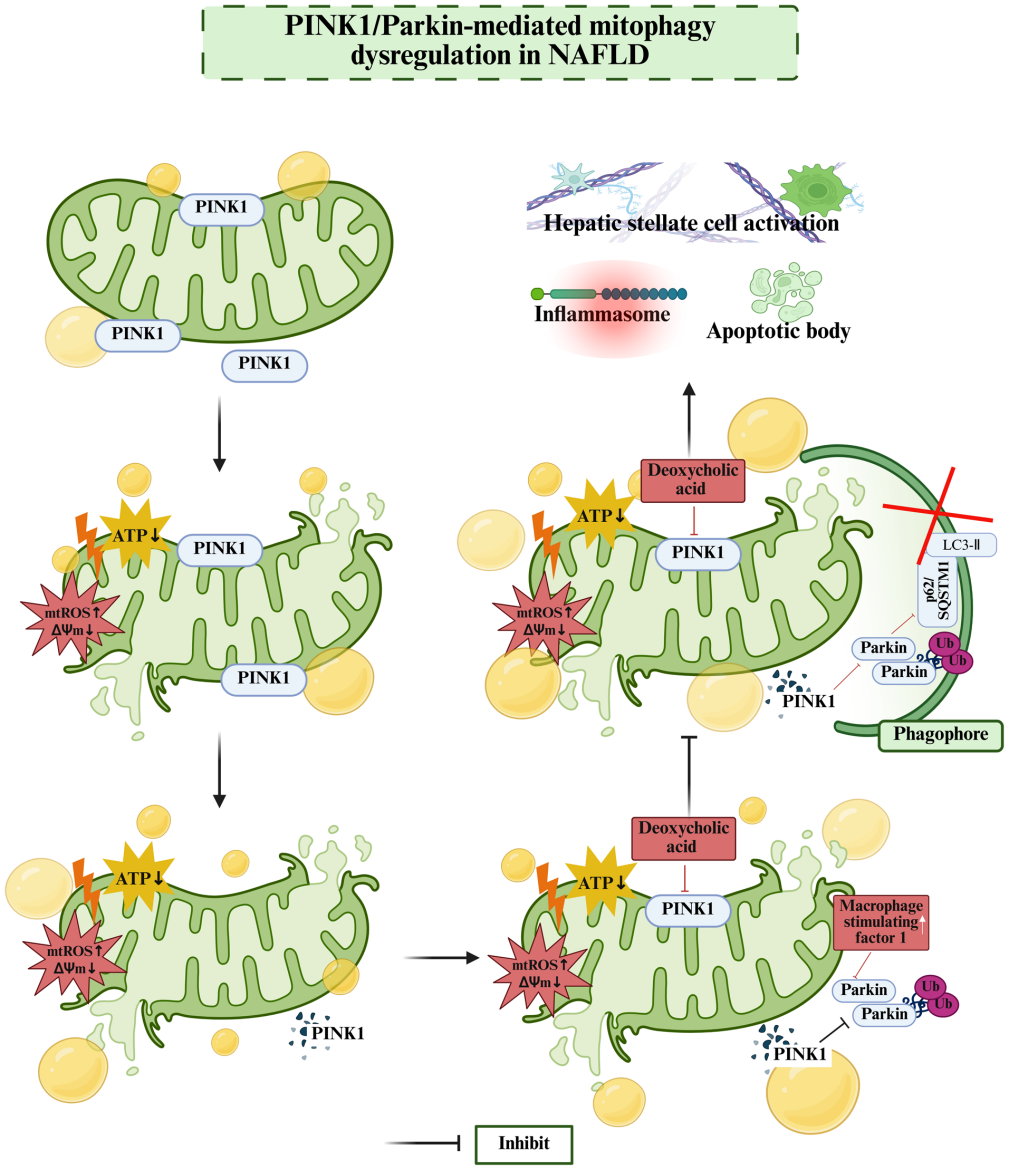
Molecular mechanism of PINK1/Parkin pathway-mediated mitophagy in NAFLD. Mitochondrial-localized PINK1 senses depolarized mitochondria, and Parkin, an E3 ubiquitin ligase, participates in the mitophagy process. In the context of NAFLD, perturbations in the PINK1/Parkin pathway impede mitophagy. This triggers a series of downstream events, including the activation of inflammasomes, the formation of apoptotic bodies and the activation of hepatic stellate cells. The subsequent morphological and functional changes of mitochondria and the downstream pathological consequences, collectively demonstrate how the dysregulation of PINK1/Parkin-mediated mitophagy contributes to the progression of NAFLD. This figure was created using Biorender software (https://app.biorender.com/). PINK1, PTEN-induced putative kinase 1; NAFLD, non-alcoholic fatty liver disease; mtROS, mitochondrial reactive oxygen species; ΔΨm, mitochondrial membrane potential; LC3-II, membrane-bound microtubule-associated protein 3; p62/SQSTM1, sequestosome 1; Ub, ubiquitin.

An important upstream regulator of mitophagy is macrophage stimulating factor 1, which is significantly upregulated in NAFLD models and inhibits Parkin-mediated mitophagy, thereby exacerbating the progression of NAFLD (60). A study of mice with liver-specific Parkin gene knockout (LKO) revealed that HFD feeding induced a 45% increase in hepatic steatosis, along with significant reductions in mitochondrial respiratory capacity and efficiency. In addition, lipid metabolism and fibrogenic pathways were dysregulated in the HFD-fed LKO mice, even when fed with a high-fiber diet. These findings suggest that Parkin is critical for maintaining hepatic mitochondrial homeostasis and protecting against liver steatosis (61). In experimental models of NASH-driven liver fibrosis, PINK1-dependent mitophagy is similarly suppressed (62). LC3 is an established marker for autophagosomes, since it is among the earliest mammalian proteins to accumulate on autophagosomal membranes. Mechanistic evidence has shown that Parkin potentiates the function of PINK1 in oleic acid-treated cells, thereby promoting autophagosome biogenesis, as evidenced by LC3 aggregation on autophagosomal membranes. Correspondingly, study of the PINK1/Parkin-mediated mitophagy pathway has shown that reduced expression of PINK1 or Parkin exacerbates mitochondrial damage in NAFLD models, whereas increased mitophagy alleviates disease progression (63). These findings support the conclusion that NAFLD is closely associated with mitophagy regulated by the PINK1/Parkin signaling pathway (Fig. 1).

### Mitophagy driven by the PI3K/AKT/mTOR pathway and its role in NAFLD

The PI3K/AKT/mTOR pathway plays a crucial role in the regulation of cell growth and metabolism. This signaling system is actively involved in responses to insulin-like growth factor, amino acid sensing, cancer therapy and autophagy. Preclinical investigations have also revealed that the PI3K/AKT signaling pathway is a key contributor to hepatic steatosis, fatty liver pathogenesis and fibrogenic progression (64). Activation of this pathway has been shown to notably reduce the expression of liver fibrosis markers and clearly improve liver health (65). Therefore, the modulation of mitophagy by manipulation of the PI3K/AKT/mTOR-dependent signaling pathway to eliminate damaged mitochondria may be a potential therapeutic approach for the management of NAFLD (66).

### Mitophagy driven by the AMPK pathway and its role in NAFLD

**Figure 2.**
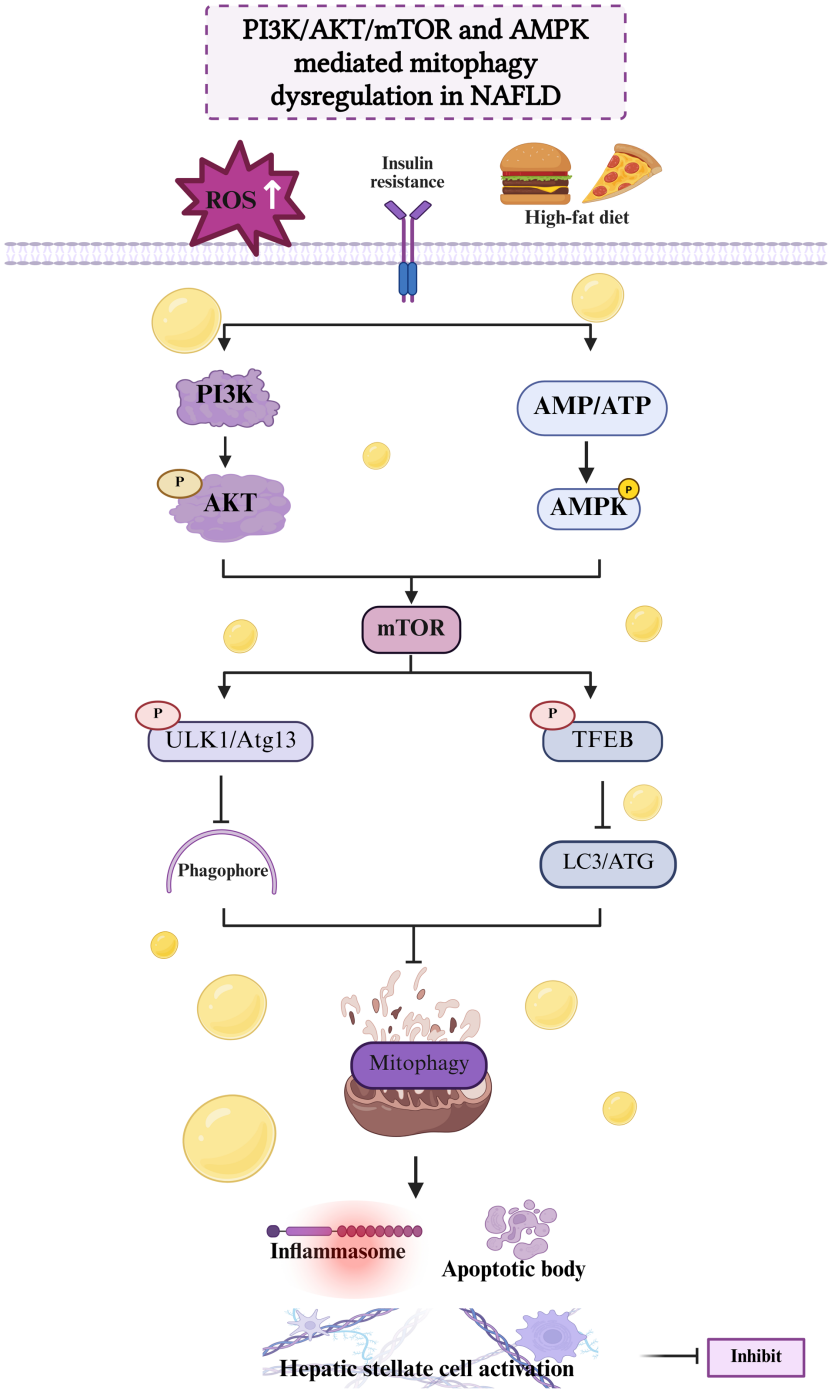
Molecular mechanism of PI3K/AKT/mTOR and AMPK pathway-mediated mitophagy in NAFLD. In the development of NAFLD, the PI3K/AKT/mTOR and AMPK signaling pathways are perturbed and ROS levels are elevated due to a high-fat diet and insulin resistance. The PI3K/AKT pathway activates mTOR, while AMPK responds to AMP/ATP changes to influence mTOR. Dysregulated mTOR impacts ULK1/Atg13 and TFEB, disrupting phagophore formation and mitophagy. Impaired mitophagy triggers inflammasome activation, apoptotic bodies and hepatic stellate cell activation, exacerbating NAFLD progression. This figure was created using Biorender software (https://app.biorender.com/). AMPK, AMP-activated protein kinase; NAFLD, non-alcoholic fatty liver disease; ROS, reactive oxygen species; ULK1, Unc-51 like autophagy activating kinase 1; Atg13, autophagy-related protein 13; TFEB, transcription factor EB; LC3, microtubule-associated protein 3; ATG, autophagy-related genes; P, phosphorylation.

AMPK is a fundamental regulator of cellular energy homeostasis. Activated by upstream kinases, AMPK senses the cellular energy status and modulates cell metabolism and growth accordingly. The major functions of the AMPK pathway include the regulation of metabolism and preservation of the cellular energy balance (67). A study has demonstrated that the AMPK-mediated regulation of ubiquinol-cytochrome *c* reductase core protein 2 is a key mitochondrial event in the mitophagy process (68). Furthermore, phosphorylation of the AMPKβ1 subunit has been found to be essential for promoting autophagy and preserving mitochondrial homeostasis under conditions of elevated fatty acid exposure (69). AMPK has been shown to reduce the production of cholesterol and fats while promoting autophagy and fatty acid oxidation, thereby exerting a regulatory effect on cellular metabolism (70–72). Research has also shown that AMPK reduces hepatic lipogenesis and increases fatty acid oxidation via the modulation of upstream like liver kinase B and downstream molecules such as sterol regulatory element-binding protein 1c (SREBP-1c), acetyl-CoA carboxylase, ULK1, etc. This regulatory process contributes to the regulation of mitochondrial autophagy, which plays a key role in the pathogenesis of NASH and liver fibrosis (73) (Fig. 2).

### Mitophagy driven by other pathways and its roles in NAFLD

In addition to the aforementioned classical mitochondrial autophagy regulatory pathways, other more recently identified receptors and nonclassical pathways also regulate mitophagy and influence the progression of NAFLD. One such receptor is FUN14 domain-containing protein 1 (FUNDC1), a mitochondrial outer membrane protein regulated by fatty acid metabolism. Palmitic acid (PA) metabolites bind to FUNDC1 homodimers, promoting their depolymerization and degradation, which suppresses mitophagy and exacerbates NAFLD. In NASH mouse models, reduced expression of FUNDC1 results in the accumulation of damaged mitochondria, increased ROS production and activation of the NLRP3 inflammasome, all of which exacerbate hepatic steatosis (74). Similarly, in cadmium-induced NAFLD in adolescent rats fed a HFD, aggravated liver damage accompanied by further activation of the NLRP3 inflammasome was observed. In addition, cadmium inhibited the mitochondrial autophagy receptor FUNDC1, leading to the dysregulation of mitochondrial dynamics, increased ROS production and the aggravation of NAFLD (75).

Prohibitin 2 (PHB2), a mitochondrial inner membrane protein essential for the maintenance of mitochondrial structure and function, has been found its deletion in hepatocytes leads to mitochondrial fragmentation, disrupted lipid and glucose metabolisms, and increased liver triglyceride accumulation, which impairs mitophagy, disrupts mitochondrial function, and aggravates liver steatosis and inflammation, and is closely associated with the occurrence and development of NAFLD (76).

Lipid signaling molecules also play pivotal roles in non-classical pathways involved in NAFLD. For example, ceramide (CER) levels in very low-density lipoprotein (VLDL) particles from insulin-resistant men have been found to correlate with the number of VLDL particles (77). CERs can target autophagosomes to mitochondria, inducing lethal mitophagy, which may contribute to the progression of NAFLD (78). Additionally, noncoding RNAs, such as circular RNAs (circRNAs) and microRNAs (miRNAs/miRs), regulate mitophagy core molecules and signaling pathways, thereby influencing NAFLD development (79,80).

## 5. Targeting mitophagy and autophagy in the treatment of NAFLD

**Figure 3.**
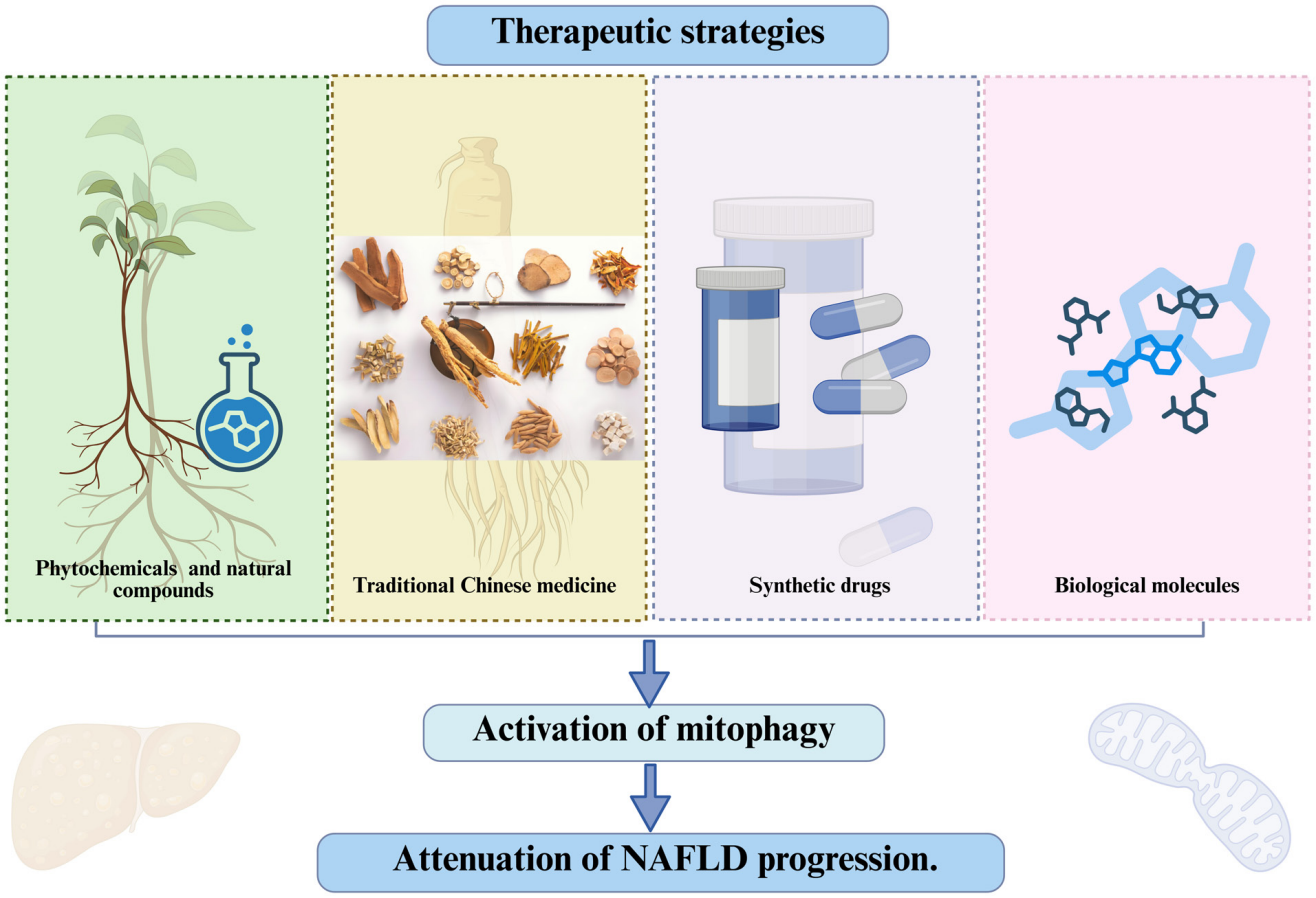
Therapeutic interventions for NAFLD based on mitophagy. An outline of therapeutic strategies for NAFLD that target mitophagy to slow disease progression. The four types of interventions are plant monomers and natural compounds, traditional Chinese medicine, synthetic drugs and biological molecules. These approaches aim to activate mitophagy, a key process for clearing damaged mitochondria, ultimately delaying the advancement of NAFLD. This figure was created using Biorender software (https://app.biorender.com/). NAFLD, non-alcoholic fatty liver disease.

It is evident that mitophagy plays an important role in the development of NAFLD. Therefore, restoring or promoting mitophagy may be a promising therapeutic strategy for this disease. A review of the literature review reveals that traditional Chinese medicines (TCMs), which are considered valuable treatment options in China, can ameliorate NAFLD by modulating mitochondrial autophagy. By exploring this topic, the present review aims to offer fresh perspectives on the therapeutic approaches useful to address the dynamic progression from liver steatosis to NASH and liver fibrosis (Fig. 3, Table I).

### Therapeutic approaches for NAFLD involving mitophagy regulation via the PINK1/Parkin pathway

Induction of the PINK1/Parkin-mediated pathway helps to repair mitochondrial damage; therefore, activating this pathway may slow the course of NAFLD. In the early steatosis stage, hepatic lipid deposition triggers mitophagy to eliminate damaged mitochondria and reduce the accumulation of fat. Sesamin, a lignan compound found in sesame seeds and oil, has been shown to restore defective mitophagy via the PINK1/Parkin pathway, reducing PA-induced lipid buildup in HepG2 cells, which suggests its promise in the prevention of hepatic steatosis (81). Similarly, hesperidin, a bioactive citrus flavonoid, interferes in fatty liver disease by increasing the activity of the PINK1/Parkin pathway to clear dysfunctional mitochondria, thereby restoring PA-impaired mitochondrial function and supporting cell homeostasis (82). Novel sesquiterpenes and *Penicillium*-derived viral toxins have also been reported to alleviate lipid accumulation and ameliorate NAFLD by regulating PINK1/Parkin-mediated mitophagy (83).

One of the main bioactive compounds in extra virgin olive oil is hydroxytyrosol (HT). In one study, an HFD was fed to Japanese seabass (*Lateolabrax maculates*) to create a model of NAFLD, with subsequent analysis showing reduced growth, increased liver fat accumulation, impaired mitochondrial function, oxidative stress and downregulated mitophagy-related genes, which were alleviated by HT via mitophagy. In addition, *in vitro* assays using liver cell lines revealed that HT inhibited fat accumulation and oxidative stress by triggering mitophagy via the PINK1 pathway (84).

Since NASH is characterized by severe inflammation and oxidative stress, the inhibition of inflammasomes and restoration of mitochondrial function are critical therapeutic goals. Cyanidin-3-O-glucoside (C3G), an anthocyanin of the flavonoid family, was found by Li *et al* (85) to increase PINK1 and Parkin expression levels and their mitochondrial location in PA-treated AML-12 cells and HFD-induced mouse models. In addition to improving systemic glucose metabolism, C3G also promoted PINK1-mediated mitophagy, facilitating the elimination of damaged mitochondria, reducing hepatic oxygen consumption, suppressing activation of the NLRP3 inflammasome and attenuating liver steatosis.

Corn peptide is a small peptide extracted from corn that possesses numerous biological properties, including antioxidant and anti-obesity effects. Yao *et al* (86) established an NAFLD cell model using FFA-induced HepG2 cells and demonstrated that corn peptide upregulated the expression of mitophagy-related proteins including PINK1, Parkin, ATG7 and LC3I/II, enhanced the levels of fatty acid β-oxidation-associated proteins such as peroxisome proliferator-activated receptor α and peroxisome proliferator-activated receptor gamma coactivator 1α and downregulated lipogenesis-related proteins such as SREBP-1c and fatty acid synthase. Therefore, it led to a reduction in p62 levels, alleviated cellular injury, restored mitochondrial function and reduced lipid accumulation via activation of the PINK1/Parkin pathway mitophagy. In addition, the TCM formulation Yang-Gan-Jiang-Mei has been shown to promote PINK1/Parkin-dependent mitophagy, suppress NLRP3 inflammasome activation and restore mitochondrial function, thereby alleviating NASH (87).

In liver fibrosis, excessive extracellular matrix deposition and activation of hepatic stellate cells are key pathological features; mitochondria-targeted ubiquinone alleviates these features and the development of hepatic fibrosis by activating PINK1/Parkin-mediated mitophagy (88). Similarly, furin, a key eukaryotic endonuclease, has been shown to inhibit the activation of murine hepatic stellate cells and ameliorate fibrosis via the same mitophagy pathway (89). Collectively, these findings highlight the pivotal role of PINK1/Parkin-mediated mitochondrial autophagy in NAFLD pathogenesis and support its potential as a therapeutic target in the progression of NAFLD from steatosis to fibrosis.

### Therapeutic approaches for NAFLD involving mitophagy regulation via the PI3K/AKT/mTOR pathway

Prebiotics may contribute to the treatment of NAFLD during the steatosis stage by promoting mitophagy via the PI3K/AKT/mTOR-mediated signaling pathway. Similarly, several phytochemicals, including anthocyanins, have been shown to promote autophagy with strong potential anti-NAFLD effects. For example, anthocyanins have been demonstrated to accomplish this by activating the AMPK/mTOR pathway (66,90). Network analysis and experimental verification have demonstrated that luteolin, the primary bioactive metabolite of *Salvia miltiorrhiza* Bunge*-Reynoutria japonica* Houtt, can induce mitophagy via this pathway, thereby alleviating NAFLD (91). Flavonoids derived from *Pueraria radix*, the main active components of this herb and common ingredients in TCM formulations, also activate the PI3K/AKT/mTOR pathway to trigger mitophagy, thereby reducing hepatic lipid accumulation and inflammation in NAFLD (92).

Acute-on-chronic liver failure (ACLF), whether explicitly recognized or not, is characterized by the abrupt deterioration of pre-existing chronic liver disease (93). According to a recent population-based analysis conducted between 2006 and 2014, the most prevalent underlying etiology of all hospitalizations for ACLF was NASH (94). In a recent study, Jianpi Lishi Yanggan formula (YGF) was used to treat a mouse model of ACLF and an *in vitro* model of hepatocyte injury induced by D-galactosamine/lipopolysaccharide. The results indicated that YGF promoted mitophagy by blocking the PI3K/AKT signaling pathway, thereby mitigating liver cell damage and reducing the hepatic inflammatory response in the ACLF model mice (95).

Liver fibrosis is essentially an aberrant repair response to chronic liver injury. When fibrosis persist, it progresses to cirrhosis, a major risk factor for HCC, the most advanced stage in the spectrum of NAFLD and a serious threat to health. Mallotucin D (MLD), a clerodane diterpenoid derivative, has been demonstrated to induce mitophagy by promoting the production of mitochondria-derived ROS and blocking the PI3K/AKT/mTOR pathway. As a result, MLD is emerging as a potential therapeutic agent for HCC (96). Notably, the Chong-Lou-Yao-Fang formulation, used as an Naxi ethnic TCM formulation for liver cancer, and aloperine extracted from *Sophora alopecuroides* have both been found to suppress HCC by modulating PI3K/AKT signaling, thereby reducing the mitochondrial membrane potential in hepatocytes. Given that mitochondrial depolarization is a key event in the initiation of mitophagy, these findings suggests that the autophagic clearance of damaged mitochondrial may contribute to their therapeutic effects (97,98).

### Therapeutic approaches for NAFLD involving mitophagy regulation via the AMPK pathway

The potential of AMPK signaling pathway-mediated mitophagy in the management of NAFLD has been explored in numerous studies. For example, in one study, an inducible knockout murine model was generated by knocking out the two AMPKβ subunits in adipocytes. Examination of this iβ1β2AKO model indicated that the brown adipose tissue of the iβ1β2AKO mice exhibited disruptions in mitophagy-associated markers. This implies that reduced AMPK activity in adipose tissue impairs mitophagy, which subsequently compromises mitochondrial function in both white and brown fat tissues (99). These novel findings suggest that the enhancement of mitochondrial and AMPK function in obese tissue may be an effective strategy to decrease the incidence of NAFLD (100).

Quercetin, a natural flavonoid widely distributed in plants and fruits, has been shown to enhance mitophagy in mouse models of NAFLD. Specifically, quercetin mitigates NAFLD via AMPK-mediated mitophagy, thereby improving lipid metabolism (101). Shenling Baizhu powder is a classical formulation that in TCM is thought to strengthen the spleen and replenish qi. This formulation has been shown to reduce hepatic lipid accumulation and modulate mitochondrial bioenergetics and mitophagy through the uncoupling protein 2/AMPK/ATPase inhibitory factor 1 signaling axis in preclinical NAFLD models (102). In addition, solute carrier family 7 member 11 has been reported to prevent AMPK-regulated mitophagy and the subsequent activation of the NLRP3 inflammasome, thereby decreasing the incidence of NASH-associated inflammation (103). Similarly, JT003 (an adiponectin receptors 1/2 agonist) and V14 (an inhibitor of the elastin-derived peptides-elastin binding protein interaction), together act as AMPK activators, have been shown act in concert to ameliorate NASH and liver fibrosis by promoting autophagy and increasing antioxidant capacity, along with their ability to exert regulatory effects in mitochondria via AMPK (104).

### Treatment of NAFLD by targeting other mitophagy pathways

*Gentianella acuta* is traditionally used in TCM for the treatment of jaundice and hepatitis (105). Certain xanthone-type flavonoids extracted from this herb have demonstrated therapeutic potential. For example, in one study, 7′-hydroxyl-substituted xanthones were found to promote FUNDC1-mediated mitophagy, thereby alleviating hepatic steatosis in obese diabetic mice (106). Also, in HCC model mice, the knockout of FUNDC1 in hepatocytes was demonstrated to exacerbate diethylnitrosamine-induced HCC due to the accumulation of dysfunctional mitochondria, suggesting that FUNDC1-mediated mitophagy and its role in the regulation of inflammation may offer therapeutic benefits for HCC (107).

As a first-line therapy for HCC, sorafenib is often limited by drug resistance. Notably, the artemisinin derivative artemether has been shown to counteract sorafenib resistance in HCC by enhancing FUNDC1-mediated mitophagy, suggesting a potential strategy to improve therapeutic efficacy (108). Formononetin, an isoflavone, protects the liver via PHB2-mediated mitophagy (109). In addition, CER levels are elevated in NAFLD, and the NASH therapeutic drug pioglitazone reduces specific CER subtypes, thereby improving hepatic mitochondrial oxidative function and alleviating NASH (110).

CircRNAs act as molecular sponges for miRNAs, and indirectly modulate mitophagy pathways by sequestering specific miRNAs and reducing their ability to inhibit the expression of target genes, a mechanism central to NAFLD progression. For example, hsa_circ_0048179 alleviates oleate/palmate-induced lipid overload and mitochondrial crista disruption by competitively binding to miR-188-3p in hepatocytes (111). In addition, circ608 enhances PINK1-mediated mitophagy in hepatic stellate cells via the inhibition of miR-222, thereby ameliorating NASH-associated liver fibrosis (62).

## 6. Summary and prospects

**Figure 4.**
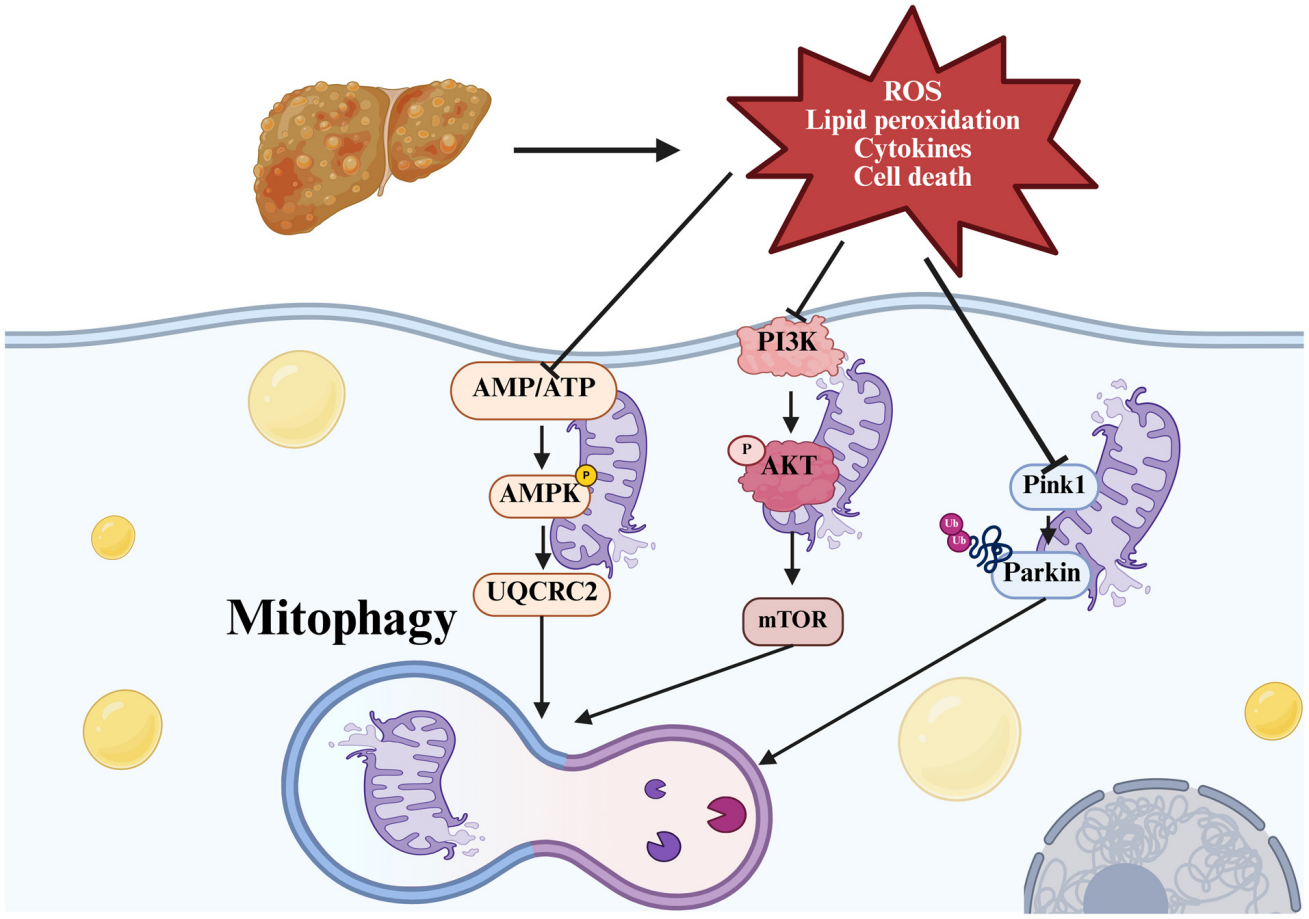
Graphical abstract summarizing the mechanism of mitophagy in NAFLD. ROS and cytokine production, lipid peroxidation and cell death contribute to mitophagy in NAFLD. The PINK1/Parkin, PI3K/AKT/mTOR and AMPK pathways are the main signaling pathways involved in the regulation of mitophagy. This figure was created using Biorender software (https://app.biorender.com/). NAFLD, non-alcoholic fatty liver disease; ROS, reactive oxygen species; PINK1, PTEN-induced putative kinase 1; AMPK, AMP-activated protein kinase; UQCRC2, ubiquinol-cytochrome *c* reductase core protein 2; P, phosphorylation.

In conclusion, as the prevalence of obesity and concomitant metabolic diseases increases, NAFLD has emerged as a major cause of chronic liver disease in developed regions of Europe, America and China. NAFLD represents a dynamic pathological continuum ranging from simple steatosis to NASH and liver fibrosis, with mitochondrial dysfunction playing a pivotal detrimental role in all stages of the disease. The stimulation of mitophagy has been identified as a promising therapeutic strategy, with the potential to alleviate pathological manifestations and slow the progression of NAFLD. The present review has outlined the impact of three classical signaling pathways and emerging regulatory factors on NAFLD (Fig. 4), prompting the proposal that integrating pharmacological interventions with natural remedies, particularly TCMs, holds unique therapeutic value. Notably, Shenling Baizhu powder, a classical formulation from the Song Dynasty has demonstrated promising clinical efficacy in the management of NAFLD (102). In addition, Shugan Xiaozhi formula, which is considered a cornerstone in NASH therapy, alleviates NASH by modulating mitophagy (112), and Longdan Xiegan Tang inhibits liver fibrosis via the activation of Parkin-mediated mitophagy (113). These TCM formulations offer distinct advantages, combining clinical efficacy with minimal side effects, and are innovative approaches for the management of NAFLD. Further elucidation of the molecular mechanisms underlying these classical remedies, to identify the signaling pathways and specific targets involved, may substantially enhance their therapeutic potency and long-term effectiveness: Through pinpointing which active components in the TCM act on critical NAFLD-related pathways, such as those governing hepatic lipid accumulation, mitochondrial function or fibrosis progression, researchers can prioritize or enrich these components, reducing reliance on inert or less impactful ingredients. This refinement directly boosts potency by focusing the formulation's effects on biologically relevant processes. Meanwhile, NAFLD pathogenesis varies across individuals, and by mapping how TCM components interact with specific molecular targets, clinicians can tailor dosages to a patient's molecular profile, avoiding suboptimal dosing that limits efficacy or excessive dosing that increases side effects. This precision enhances both short-term potency and long-term tolerability. Also, researchers can establish objective biomarkers to track treatment response through linking TCM efficacy to measurable molecular endpoints (e.g. enhanced mitophagy via PINK1/Parkin activation); this not only validates the formulation's biological activity, but also supports regulatory approval and standardized production, a critical step for ensuring consistent long-term effectiveness in broader patient populations.

Basic research has also led to unexpected findings with clinical relevance. For example, sorafenib, a first-line therapy for HCC, is often limited by drug resistance. A study revealed that ketoconazole, a broad-spectrum antifungal agent, activates PINK1/Parkin-mediated mitophagy by downregulating cyclooxygenase-2, and exerts a synergistic inhibitory effect on HCC when combined with ketoconazole, offering a potential strategy to overcome sorafenib resistance (114). Thyroid hormone (TH) shows therapeutic potential in NAFLD through the activation of mitophagy, thereby alleviating hepatic steatosis and liver fibrosis. Epidemiological analysis has shown an increased prevalence of NAFLD in patients with hypothyroidism, and mechanistic experiments have demonstrated that TH exerts antifibrotic effects by enhancing mitochondrial function and mitophagy (115). In a study of 20 euthyroid men with type 2 diabetes and NAFLD, low-dose thyroxine treatment reduced the intrahepatic lipid content from baseline by 12% (± SEM, 26%). This SEM indicates considerable inter-individual variability: While 15 out of 20 patients exhibited a decrease, notable age-dependent differences were observed (≥50 years: −23±6%; <50 years: 2±8%, P=0.024). Additionally, the small sample size of 20 may have amplified the impact of individual variations on the SEM, compromising statistical robustness and underscoring the necessity for targeted patient selection, such as identifying potential beneficiaries based on age or other biomarkers (116). This finding prompted the evaluation of resmetirom (MGL-3196), an oral selective TH receptor β1 (THRβ1) agonist, in NASH trials, where it significantly reduced hepatic fat content and achieved >2-point reductions in NASH activity scores compared with those in patients treated with placebo (117,118). An advanced formulation of resmetirom, known as Rezdiffra, was approved by the US Food and Drug Administration in 2024 as the first drug for NASH and metabolic dysfunction-associated steatohepatitis, exemplifying how mechanistic insights into TH-mediated mitophagy can drive drug development (119). These cases underscore how basic research can uncover unexpected therapeutic synergies and novel agents, providing a scientific basis for clinical translation to ease patient burden. By elucidating molecular mechanisms, such as TH-induced mitophagy, advancements in pharmaceutical innovations resulting in increased therapeutic efficacy can be achieved.

Notably, mitophagy appears to play complex roles in advanced NAFLD. During liver fibrosis, it can activate hepatic stellate cells to promote fibrogenesis (120), and certain compounds demonstrate anti-HCC effects by inhibiting mitophagy (121). However, mitophagy has also been associated with the development of sorafenib resistance (122). Current research in these areas remains insufficient, indicating the importance of further in-depth exploration.

Future investigations integrating multiomics approaches and precision medicine strategies hold promise for the development of comprehensive therapeutic modalities to target mitophagy in NAFLD. Furthermore, the clinical translation of innovative agents such as THRβ agonists, along with the optimization of TCM formulations based on the ‘syndrome differentiation and treatment’ principle will broaden therapeutic horizons (123), paving the way for safer and more efficacious interventions.

## Figures and Tables

**Table I.** Therapeutic interventions for non-alcoholic fatty disease based on mitophagy.

First author, year	Therapies	Targeted signaling pathways	Effects	(Refs.)
Xu *et al*, 2022	Circ608	Enhances PINK1-mediated mitophagy via miR-222 inhibition	Ameliorates NASH-associated liver fibrosis	(62)
Tsuji *et al*, 2023	Prebiotics	PI3K/AKT/mTOR	Eliminates impaired mitochondria	(66)
Dong *et al*, 2024	Sesamin	PINK1/Parkin	Reduces PA-induced lipid build-up	(81)
Li *et al*, 2024	Hesperidin	PINK1/Parkin	Restores PA-impaired mitochondrial function and maintains cell balance	(82)
Zhang *et al*, 2024	Novel sesquiterpenes and *Penicillium*-derived viral toxins	PINK1/Parkin	Alleviates lipid accumulation	(83)
Dong *et al*, 2022	Hydroxytyrosol	PINK1/Parkin	Inhibits fat accumulation and oxidative stress	(84)
Li *et al*, 2020	Cyanidin-3-O-glucoside	PINK1/Parkin	Removes impaired mitochondria, reduces liver oxygen consumption, activates the NLRP3 inflammasome and lessens liver steatosis	(85)
Yao *et al*, 2023	Corn peptide	PINK1/Parkin	Rectifies mitochondrial malfunction and decreases lipid build-up	(86)
Wu *et al*, 2024	Yang-Gan-Jiang-Mei	PINK1/Parkin	Suppresses NLRP3 inflammasome activation, and restores mitochondrial function to alleviate NASH	(87)
Song *et al*, 2025	Furin	PINK1/Parkin	Inhibits mouse hepatic stellate cell activation and ameliorates fibrosis	(89)
Mehmood *et al*, 2024;	Anthocyanins	AMPK/mTOR	Ameliorates mitochondrial damage	(90)
Tsuji *et al*, 2023	*Salvia miltiorrhiza*	PI3K/AKT/mTOR	Reduces fat accumulation and	(90)
Mehmood *et al*, 2024	Bunge-*Reynoutria japonica* Houtt		intracellular triglyceride levels	
Sarin *et al*, 2019	Flavonoids	PI3K/AKT/mTOR	Reduces lipid build-up and inflammation	(93)
Li *et al*, 2024	Jianpi Lishi Yanggan formula	PI3K/AKT/mTOR	Reduces liver cell damage and the liver inflammatory response in ACLF model mice	(95)
Dai *et al*, 2022	Mallotucin D	PI3K/AKT/mTOR	Promotes the production of mitochondrial ROS	(96)
Yan *et al*, 2024	Chong-Lou-Yao-Fang	PI3K/AKT	Decreases mitochondrial membrane potential	(97)
Cao *et al*, 2023	Quercetin	AMPK	Improves lipid metabolism	(101)
Yao *et al*, 2023	Shenling Baizhu powder	UCP2/AMPK/IF1	Decreases hepatic lipid accumulation and modulates mitochondrial bioenergetics and mitophagy	(102)
Lv *et al*, 2024	Solute carrier family 7 member 11	AMPK	Prevents NLRP3 inflammasome activation	(103)
Song *et al*, 2023	JT003 and V14	AMPK	Increases autophagy and antioxidant capacity	(104)
Li *et al*, 2025	7′-Hydroxyl substituted xanthones from *Gentianella acuta*	FUNDC1	Alleviates hepatic steatosis	(106)
Ma, 2024	Artemisinin derivative artemether	FUNDC1	Counteracts sorafenib resistance	(108)
Ma, 2022	Formononetin	PHB2/PINK1/Parkin	Protects the liver from ischemia/reperfusion induced injury	(109)
Kalavala-palli *et al*, 2018	Pioglitazone	Ceramide	Improves hepatic mitochondrial oxidative function	(110)
Yang *et al*, 2020	Hsa_circ_0048179	Sponges miR-188-3p	Induces lipid overload and mitochondrial crispa disruption	(111)

PINK1, PTEN-induced putative kinase 1; PA, palmitic acid; NLRP3, Nod-like receptor family pyrin domain containing 3; AMPK, AMP-activated protein kinase; NASH, non-alcoholic steatohepatitis; ACLF, acute-on-chronic liver failure; UCP2, uncoupling protein 2; IF1, ATPase inhibitory factor 1; FUNDC1, FUN14 domain-containing protein 1; ROS, reactive oxygen species.

## Data Availability

Not applicable.

## Abbreviations

NAFLD: non-alcoholic fatty liver disease
NASH: non-alcoholic steatohepatitis
NAFL: non-alcoholic fatty liver
Insulin resistance: IR
FUNDC1: FUN14 domain-containing protein 1
PHB2: prohibitin 2
TCM: traditional Chinese medicine
HCC: hepatocellular carcinoma
ER: endoplasmic reticulum
LDs: lipid droplets
ROS: reactive oxygen species
LC3: microtubule-associated protein 3
p62: sequestosome 1
HFD: high-fat diet
FFA: free fatty acid
PINK1: PTEN-induced putative kinase 1
AMPK: AMP-activated protein kinase
NLRP3: Nod-like receptor family pyrin domain containing 3
LKO: liver-specific Parkin gene knockout
CERs: ceramides
VLDL: very low-density lipoprotein
circRNAs: circular RNAs
miRNA/miR: microRNA
PA: palmitic acid
HT: hydroxytyrosol
C3G: cyanidin-3-O-glucoside
MLD: mallotucin D
YGF: Jianpi Lishi Yanggan formula
ACLF: acute-on-chronic liver failure
TH: thyroid hormone
HRβ1: TH receptor β1
SREBP-1c: sterol regulatory element-binding protein 1c

## References

[1] Raza S, Rajak S, Upadhyay A, Tewari A, Anthony Sinha R (2021). Current treatment paradigms and emerging therapies for NAFLD/NASH. Front Biosci (Landmark Ed).

[2] Eslam M, Newsome PN, Sarin SK, Anstee QM, Targher G, Romero-Gomez M, Zelber-Sagi S, Wai-Sun Wong V, Dufour JF, Schattenberg JM (2020). A new definition for metabolic dysfunction-associated fatty liver disease: An international expert consensus statement. J Hepatol.

[3] Rinella ME, Lazarus JV, Ratziu V, Francque SM, Sanyal AJ, Kanwal F, Romero D, Abdelmalek MF, Anstee QM, Arab JP (2023). A multisociety Delphi consensus statement on new fatty liver disease nomenclature. J Hepatol.

[4] Younossi ZM, Golabi P, Paik JM, Henry A, Van Dongen C, Henry L (2023). The global epidemiology of nonalcoholic fatty liver disease (NAFLD) and nonalcoholic steatohepatitis (NASH): A systematic review. Hepatology.

[5] Lu R, Liu Y, Hong T (2023). Epidemiological characteristics and management of nonalcoholic fatty liver disease/nonalcoholic steatohepatitis in China: A narrative review. Diabetes Obes Metab.

[6] Wei S, Wang L, Evans PC, Xu S (2024). NAFLD and NASH: Etiology, targets and emerging therapies. Drug Discov Today.

[7] Rong L, Zou J, Ran W, Qi X, Chen Y, Cui H, Guo J (2023). Advancements in the treatment of non-alcoholic fatty liver disease (NAFLD). Front Endocrinol (Lausanne).

[8] Qian H, Chao X, Williams J, Fulte S, Li T, Yang L, Ding WX (2021). Autophagy in liver diseases: A review. Mol Aspects Med.

[9] Ma X, McKeen T, Zhang J, Ding WX (2020). Role and mechanisms of mitophagy in liver diseases. Cells.

[10] Ramanathan R, Ali AH, Ibdah JA (2022). Mitochondrial dysfunction plays central role in nonalcoholic fatty liver disease. Int J Mol Sci.

[11] Petrescu M, Vlaicu SI, Ciumărnean L, Milaciu MV, Mărginean C, Florea M, Vesa ȘC, Popa M (2022). Chronic inflammation-A link between nonalcoholic fatty liver disease (NAFLD) and dysfunctional adipose tissue. Medicina (Kaunas).

[12] Tarantino G, Citro V, Balsano C (2021). Liver-spleen axis in nonalcoholic fatty liver disease. Expert Rev Gastroenterol Hepatol.

[13] Leung C, Rivera L, Furness JB, Angus PW (2016). The role of the gut microbiota in NAFLD. Nat Rev Gastroenterol Hepatol.

[14] Mundi MS, Velapati S, Patel J, Kellogg TA, Abu Dayyeh BK, Hurt RT (2020). Evolution of NAFLD and its management. Nutr Clin Pract.

[15] Paternostro R, Trauner M (2022). Current treatment of non-alcoholic fatty liver disease. J Intern Med.

[16] Gao Y, Zhang W, Zeng LQ, Bai H, Li J, Zhou J, Zhou GY, Fang CW, Wang F, Qin XJ (2020). Exercise and dietary intervention ameliorate high-fat diet-induced NAFLD and liver aging by inducing lipophagy. Redox Biol.

[17] Cao W, Li J, Yang K, Cao D (2021). An overview of autophagy: Mechanism, regulation and research progress. Bull Cancer.

[18] Chen X, Tsvetkov AS, Shen HM, Isidoro C, Ktistakis NT, Linkermann A, Koopman WJH, Simon HU, Galluzzi L, Luo S (2024). International consensus guidelines for the definition, detection, and interpretation of autophagy-dependent ferroptosis. Autophagy.

[19] Wang H, Li X, Zhang Q, Fu C, Jiang W, Xue J, Liu S, Meng Q, Ai L, Zhi X (2024). Autophagy in disease onset and progression. Aging Dis.

[20] Vargas J, Hamasaki M, Kawabata T, Youle RJ, Yoshimori T (2023). The mechanisms and roles of selective autophagy in mammals. Nat Rev Mol Cell Bio.

[21] Yang K, Yan Y, Yu A, Zhang R, Zhang Y, Qiu Z, Li Z, Zhang Q, Wu S, Li F (2024). Mitophagy in neurodegenerative disease pathogenesis. Neural Regen Res.

[22] Li W, He P, Huang Y, Li YF, Lu J, Li M, Kurihara H, Luo Z, Meng T, Onishi M (2021). Selective autophagy of intracellular organelles: Recent research advances. Theranostics.

[23] Waite KA, Burris A, Vontz G, Lang A, Roelofs J (2022). Proteaphagy is specifically regulated and requires factors dispensable for general autophagy. J Biol Chem.

[24] Hsiao PJ, Chiou HC, Jiang HJ, Lee MY, Hsieh TJ, Kuo KK (2017). Pioglitazone enhances cytosolic lipolysis, β-oxidation and autophagy to ameliorate hepatic steatosis. Sci Rep.

[25] Feng S, Sun Z, Jia X, Li L, Wu Y, Wu C, Lin L, Liu J, Zeng B (2023). Lipophagy: Molecular mechanisms and implications in hepatic lipid metabolism. Front Biosci (Landmark Ed).

[26] Zhang S, Peng X, Yang S, Li X, Huang M, Wei S, Liu J, He G, Zheng H, Yang L (2022). The regulation, function, and role of lipophagy, a form of selective autophagy, in metabolic disorders. Cell Death Dis.

[27] Laval T, Ouimet M (2023). A role for lipophagy in atherosclerosis. Nat Rev Cardiol.

[28] Robichaud S, Fairman G, Vijithakumar V, Mak E, Cook DP, Pelletier AR, Huard S, Vanderhyden BC, Figeys D, Lavallée-Adam M (2021). Identification of novel lipid droplet factors that regulate lipophagy and cholesterol efflux in macrophage foam cells. Autophagy.

[29] Pu M, Zheng W, Zhang H, Wan W, Peng C, Chen X, Liu X, Xu Z, Zhou T, Sun Q (2023). ORP8 acts as a lipophagy receptor to mediate lipid droplet turnover. Protein Cell.

[30] Chung J, Park J, Lai ZW, Lambert TJ, Richards RC, Zhang J, Walther TC, Farese RV (2023). The Troyer syndrome protein spartin mediates selective autophagy of lipid droplets. Nat Cell Biol.

[31] Zhang M, Wang Z, Zhao Q, Yang Q, Bai J, Yang C, Zhang ZR, Liu Y (2024). USP20 deubiquitinates and stabilizes the reticulophagy receptor RETREG1/FAM134B to drive reticulophagy. Autophagy.

[32] Gubas A, Dikic I (2022). ER remodeling via ER-phagy. Mol Cell.

[33] Reggiori F, Molinari M (2022). ER-phagy: Mechanisms, regulation, and diseases connected to the lysosomal clearance of the endoplasmic reticulum. Physiol Rev.

[34] Wang S, Long H, Hou L, Feng B, Ma Z, Wu Y, Zeng Y, Cai J, Zhang DW, Zhao G (2023). The mitophagy pathway and its implications in human diseases. Signal Transduct Target Ther.

[35] Doblado L, Lueck C, Rey C, Samhan-Arias AK, Prieto I, Stacchiotti A, Monsalve M (2021). Mitophagy in human diseases. Int J Mol Sci.

[36] Lu Y, Li Z, Zhang S, Zhang T, Liu Y, Zhang L (2023). Cellular mitophagy: Mechanism, roles in diseases and small molecule pharmacological regulation. Theranostics.

[37] Degli Esposti M (2024). Did mitophagy follow the origin of mitochondria?. Autophagy.

[38] Yang M, Wei X, Yi X, Jiang DS (2024). Mitophagy-related regulated cell death: Molecular mechanisms and disease implications. Cell Death Dis.

[39] Liu B, Cao Y, Wang D, Zhou Y, Zhang P, Wu J, Chen J, Qiu J, Zhou J (2021). Zhen-Wu-Tang induced mitophagy to protect mitochondrial function in chronic glomerulonephritis via PI3K/AKT/mTOR and AMPK pathways. Front Pharmacol.

[40] Poole LP, Macleod KF (2021). Mitophagy in tumorigenesis and metastasis. Cell Mol Life Sci.

[41] Allaire M, Rautou PE, Codogno P, Lotersztajn S (2019). Autophagy in liver diseases: Time for translation?. J Hepatol.

[42] An L, Wirth U, Koch D, Schirren M, Drefs M, Koliogiannis D, Niess H, Andrassy J, Guba M, Bazhin AV (2023). Metabolic role of autophagy in the pathogenesis and development of NAFLD. Metabolites.

[43] González-Rodríguez A, Mayoral R, Agra N, Valdecantos MP, Pardo V, Miquilena-Colina ME, Vargas-Castrillón J, Lo Iacono O, Corazzari M, Fimia GM (2014). Impaired autophagic flux is associated with increased endoplasmic reticulum stress during the development of NAFLD. Cell Death Dis.

[44] Li Q, Lin Y, Liang G, Xiao N, Zhang H, Yang X, Yang J, Liu A (2023). Autophagy and senescence: The molecular mechanisms and implications in liver diseases. Int J Mol Sci.

[45] Nasiri-Ansari N, Nikolopoulou C, Papoutsi K, Kyrou I, Mantzoros CS, Kyriakopoulos G, Chatzigeorgiou A, Kalotychou V, Randeva MS, Chatha K (2021). Empagliflozin attenuates non-alcoholic fatty liver disease (NAFLD) in high fat diet fed ApoE^(−/-)^ mice by activating autophagy and reducing ER stress and apoptosis. Int J Mol Sci.

[46] Chen Y, Yang F, Shi Y, Sheng J, Wang Y, Zhang L, Zhou J, Jin Y, Yan Y (2024). RNF31 alleviates liver steatosis by promoting p53/BNIP3-related mitophagy in hepatocytes. Free Radical Bio Med.

[47] Nazeer B, Khawar MB, Khalid MU, Hamid SE, Rafiq M, Abbasi MH, Sheikh N, Ali A, Fatima H, Ahmad S (2024). Emerging role of lipophagy in liver disorders. Mol Cell Biochem.

[48] Scorletti E, Carr RM (2022). A new perspective on NAFLD: Focusing on lipid droplets. J Hepatol.

[49] Grefhorst A, van de Peppel IP, Larsen LE, Jonker JW, Holleboom AG (2021). The role of lipophagy in the development and treatment of non-alcoholic fatty liver disease. Front Endocrinol (Lausanne).

[50] Zechner R, Madeo F, Kratky D (2017). Cytosolic lipolysis and lipophagy: Two sides of the same coin. Nat Rev Mol Cell Biol.

[51] Byrnes K, Blessinger S, Bailey NT, Scaife R, Liu G, Khambu B (2022). Therapeutic regulation of autophagy in hepatic metabolism. Acta Pharm Sin B.

[52] Minami Y, Hoshino A, Higuchi Y, Hamaguchi M, Kaneko Y, Kirita Y, Taminishi S, Nishiji T, Taruno A, Fukui M (2023). Liver lipophagy ameliorates nonalcoholic steatohepatitis through extracellular lipid secretion. Nat Commun.

[53] Gusdon AM, Song KX, Qu S (2014). Nonalcoholic fatty liver disease: Pathogenesis and therapeutics from a mitochondria-centric perspective. Oxid Med Cell Longev.

[54] Aryapour E, Kietzmann T (2022). Mitochondria, mitophagy, and the role of deubiquitinases as novel therapeutic targets in liver pathology. J Cell Biochem.

[55] Lee J, Park JS, Roh YS (2019). Molecular insights into the role of mitochondria in non-alcoholic fatty liver disease. Arch Pharm Res.

[56] Moore MP, Cunningham RP, Meers GM, Johnson SA, Wheeler AA, Ganga RR, Spencer NM, Pitt JB, Diaz-Arias A, Swi AIA (2022). Compromised hepatic mitochondrial fatty acid oxidation and reduced markers of mitochondrial turnover in human NAFLD. Hepatology.

[57] Chen J, Jian L, Guo Y, Tang C, Huang Z, Gao J (2024). Liver cell mitophagy in metabolic dysfunction-associated steatotic liver disease and liver fibrosis. Antioxidants (Basel).

[58] Cao Y, Chen X, Pan F, Wang M, Zhuang H, Chen J, Lu L, Wang L, Wang T (2023). Xinmaikang-mediated mitophagy attenuates atherosclerosis via the PINK1/Parkin signaling pathway. Phytomedicine.

[59] Gao X, Ruan Y, Zhu X, Lin X, Xin Y, Li X, Mai M, Guo H (2022). Deoxycholic acid promotes pyroptosis in free fatty acid-induced steatotic hepatocytes by inhibiting PINK1-mediated mitophagy. Inflammation.

[60] Zhou T, Chang L, Luo Y, Zhou Y, Zhang J (2019). Mst1 inhibition attenuates non-alcoholic fatty liver disease via reversing Parkin-related mitophagy. Redox Biol.

[61] Edmunds LR, Xie B, Mills AM, Huckestein BR, Undamatla R, Murali A, Pangburn MM, Martin J, Sipula I, Kaufman BA (2020). Liver-specific Prkn knockout mice are more susceptible to diet-induced hepatic steatosis and insulin resistance. Mol Metab.

[62] Xu ZX, Li JZ, Li Q, Xu MY, Li HY (2022). CircRNA608-microRNA222-PINK1 axis regulates the mitophagy of hepatic stellate cells in NASH related fibrosis. Biochem Biophys Res Commun.

[63] He H, Tang Y, Zhuang L, Zheng Y, Huang X (2024). PINK1/Park2-mediated mitophagy relieve non-alcoholic fatty liver disease. Physiol Res.

[64] Matsuda S, Kobayashi M, Kitagishi Y (2013). Roles for PI3K/AKT/PTEN pathway in cell signaling of nonalcoholic fatty liver disease. ISRN Endocrinol.

[65] Shamsan E, Almezgagi M, Gamah M, Khan N, Qasem A, Chuanchuan L, Haining F (2024). The role of PI3k/AKT signaling pathway in attenuating liver fibrosis: A comprehensive review. Front Med (Lausanne).

[66] Tsuji A, Yoshikawa S, Ikeda Y, Taniguchi K, Sawamura H, Morikawa S, Nakashima M, Asai T, Matsuda S (2023). Tactics with prebiotics for the treatment of metabolic dysfunction-associated fatty liver disease via the improvement of mitophagy. Int J Mol Sci.

[67] Aslam M, Ladilov Y (2022). Emerging role of cAMP/AMPK signaling. Cells.

[68] Lu X, Xuan W, Li J, Yao H, Huang C, Li J (2021). AMPK protects against alcohol-induced liver injury through UQCRC2 to up-regulate mitophagy. Autophagy.

[69] Desjardins EM, Smith BK, Day EA, Ducommun S, Sanders MJ, Nederveen JP, Ford RJ, Pinkosky SL, Townsend LK, Gutgesell RM (2022). The phosphorylation of AMPKβ1 is critical for increasing autophagy and maintaining mitochondrial homeostasis in response to fatty acids. Proc Natl Acad Sci USA.

[70] Gu M, Luo L, Fang K (2018). Crocin inhibits obesity via AMPK-dependent inhibition of adipocyte differentiation and promotion of lipolysis. Biosci Trends.

[71] Herzig S, Shaw RJ (2018). AMPK: Guardian of metabolism and mitochondrial homeostasis. Nat Rev Mol Cell Biol.

[72] Fang C, Pan J, Qu N, Lei Y, Han J, Zhang J, Han D (2022). The AMPK pathway in fatty liver disease. Front Physiol.

[73] Marcondes-de-Castro IA, Reis-Barbosa PH, Marinho TS, Aguila MB, Mandarim-de-Lacerda CA (2023). AMPK/mTOR pathway significance in healthy liver and non-alcoholic fatty liver disease and its progression. J Gastroenterol Hepatol.

[74] Chen L, Zhang Q, Meng Y, Zhao T, Mu C, Fu C, Deng C, Feng J, Du S, Liu W (2023). Saturated fatty acids increase LPI to reduce FUNDC1 dimerization and stability and mitochondrial function. EMBO Rep.

[75] Lian CY, Li HJ, Xia WH, Li Y, Zhou XL, Yang DB, Wan XM, Wang L (2024). Insufficient FUNDC1-dependent mitophagy due to early environmental cadmium exposure triggers mitochondrial redox imbalance to aggravate diet-induced lipotoxicity. Environ Pollut.

[76] Li L, Martin-Levilain J, Jiménez-Sánchez C, Karaca M, Foti M, Martinou JC, Maechler P (2019). In vivo stabilization of OPA1 in hepatocytes potentiates mitochondrial respiration and gluconeogenesis in a prohibitin-dependent way. J Biol Chem.

[77] Mucinski JM, Manrique-Acevedo C, Kasumov T, Garrett TJ, Gaballah A, Parks EJ (2020). Relationships between very low-density lipoproteins-ceramides, -diacylglycerols, and -triacylglycerols in insulin-resistant men. Lipids.

[78] Sentelle RD, Senkal CE, Jiang W, Ponnusamy S, Gencer S, Selvam SP, Ramshesh VK, Peterson YK, Lemasters JJ, Szulc ZM (2012). Ceramide targets autophagosomes to mitochondria and induces lethal mitophagy. Nat Chem Biol.

[79] Kim KM, Kim SG (2014). Autophagy and microRNA dysregulation in liver diseases. Arch Pharm Res.

[80] Yuan X, Li Y, Wen S, Xu C, Wang C, He Y, Zhou L (2022). CircLDLR acts as a sponge for miR-667-5p to regulate SIRT1 expression in non-alcoholic fatty liver disease. Lipids Health Dis.

[81] Dong M, Zhang T, Liang X, Cheng X, Shi F, Yuan H, Zhang F, Jiang Q, Wang X (2024). Sesamin alleviates lipid accumulation induced by oleic acid via PINK1/Parkin-mediated mitophagy in HepG2 cells. Biochem Biophys Res Commun.

[82] Li W, Cai Z, Schindler F, Afjehi-Sadat L, Montsch B, Heffeter P, Heiss EH, Weckwerth W (2024). Elevated PINK1/Parkin-dependent mitophagy and boosted mitochondrial function mediate protection of hepg2 cells from excess palmitic acid by hesperetin. J Agric Food Chem.

[83] Zhang H, You Y, Xu J, Jiang H, Jiang J, Su Z, Chao Z, Du Q, He F (2024). New sesquiterpenes and viridin derivatives from *Penicillium* sp. Ameliorates NAFLD by regulating the PINK1/Parkin mitophagy pathway. Bioorg Chem.

[84] Dong Y, Yu M, Wu Y, Xia T, Wang L, Song K, Zhang C, Lu K, Rahimnejad S (2022). Hydroxytyrosol promotes the mitochondrial function through activating mitophagy. Antioxidants (Basel).

[85] Li X, Shi Z, Zhu Y, Shen T, Wang H, Shui G, Loor JJ, Fang Z, Chen M, Wang X (2020). Cyanidin-3-O-glucoside improves non-alcoholic fatty liver disease by promoting PINK1-mediated mitophagy in mice. Br J Pharmacol.

[86] Yao Z, Li X, Wang W, Ren P, Song S, Wang H, Xie Y, Li X, Li Z (2023). Corn peptides attenuate non-alcoholic fatty liver disease via PINK1/Parkin-mediated mitochondrial autophagy. Food Nutr Res.

[87] Wu Y, Kuang Y, Wu Y, Dai H, Bi R, Hu J, Sun L (2024). Yang-Gan-Jiang-Mei formula alleviates non-alcoholic steatohepatitis by inhibiting NLRP3 inflammasome through mitophagy. Biotechnol Genet Eng Rev.

[88] Dou SD, Zhang JN, Xie XL, Liu T, Hu JL, Jiang XY, Wang MM, Jiang HD (2021). MitoQ inhibits hepatic stellate cell activation and liver fibrosis by enhancing PINK1/parkin-mediated mitophagy. Open Med (Wars).

[89] Song YW, Zhu YH, Ma MZ (2025). Furin inhibits HSCs activation and ameliorates liver fibrosis by regulating PTEN-L/PINK1/parkin mediated mitophagy in mouse. FASEB Bioadv.

[90] Mehmood A, Zhao L, Wang Y, Pan F, Hao S, Zhang H, Iftikhar A, Usman M (2021). Dietary anthocyanins as potential natural modulators for the prevention and treatment of non-alcoholic fatty liver disease: A comprehensive review. Food Res Int.

[91] Chen H, Yan S, Xiang Q, Liang J, Deng X, He W, Cheng Y, Yang L (2024). Network analysis and experimental verification of *Salvia miltiorrhiza* Bunge-*Reynoutria japonica* Houtt. drug pair in the treatment of non-alcoholic fatty liver disease. BMC Complement Med Ther.

[92] Sun C, Zhang J, Hou J, Hui M, Qi H, Lei T, Zhang X, Zhao L, Du H (2023). Induction of autophagy via the PI3K/Akt/mTOR signaling pathway by Pueraria flavonoids improves non-alcoholic fatty liver disease in obese mice. Biomed Pharmacother.

[93] Sarin SK, Choudhury A, Sharma MK, Maiwall R, Al Mahtab M, Rahman S, Saigal S, Saraf N, Soin AS, Devarbhavi H (2019). Acute-on-chronic liver failure: Consensus recommendations of the Asian Pacific association for the study of the liver (APASL): An update. Hepatol Int.

[94] Axley P, Ahmed Z, Arora S, Haas A, Kuo YF, Kamath PS, Singal AK (2019). NASH is the most rapidly growing etiology for acute-on-chronic liver failure-related hospitalization and disease burden in the United States: A population-based study. Liver Transpl.

[95] Li J, Huang Q, Ma W, Yi J, Zhong X, Hu R, Sun J, Ma M, Lv M, Han Z (2024). Hepatoprotective efficacy and interventional mechanism of JianPi LiShi YangGan formula in acute-on-chronic liver failure. J Ethnopharmacol.

[96] Dai X, Sun F, Deng K, Lin G, Yin W, Chen H, Yang D, Liu K, Zhang Y, Huang L (2022). Mallotucin D, a clerodane diterpenoid from Croton crassifolius, suppresses HepG2 cell growth via inducing autophagic cell death and pyroptosis. Int J Mol Sci.

[97] Yan X, Inta A, Yang X, Pandith H, Disayathanoowat T, Yang L (2024). An investigation of the effect of the traditional naxi herbal formula against liver cancer through network pharmacology, molecular docking, and in vitro experiments. Pharmaceuticals (Basel).

[98] Liu JS, Huo CY, Cao HH, Fan CL, Hu JY, Deng LJ, Lu ZB, Yang HY, Yu LZ, Mo ZX, Yu ZL (2019). Aloperine induces apoptosis and G2/M cell cycle arrest in hepatocellular carcinoma cells through the PI3K/Akt signaling pathway. Phytomedicine.

[99] Mottillo EP, Desjardins EM, Crane JD, Smith BK, Green AE, Ducommun S, Henriksen TI, Rebalka IA, Razi A, Sakamoto K (2016). Lack of adipocyte AMPK exacerbates insulin resistance and hepatic steatosis through brown and beige adipose tissue function. Cell Metab.

[100] Smith BK, Marcinko K, Desjardins EM, Lally JS, Ford RJ, Steinberg GR (2016). Treatment of nonalcoholic fatty liver disease: Role of AMPK. Am J Physiol Endocrinol Metab.

[101] Cao P, Wang Y, Zhang C, Sullivan MA, Chen W, Jing X, Yu H, Li F, Wang Q, Zhou Z (2023). Quercetin ameliorates nonalcoholic fatty liver disease (NAFLD) via the promotion of AMPK-mediated hepatic mitophagy. J Nutr Biochem.

[102] Yao Z, Guo J, Du B, Hong L, Zhu Y, Feng X, Hou Y, Shi A (2023). Effects of Shenling Baizhu powder on intestinal microflora metabolites and liver mitochondrial energy metabolism in nonalcoholic fatty liver mice. Front Microbiol.

[103] Lv T, Fan X, He C, Zhu S, Xiong X, Yan W, Liu M, Xu H, Shi R, He Q (2024). SLC7A11-ROS/αKG-AMPK axis regulates liver inflammation through mitophagy and impairs liver fibrosis and NASH progression. Redox Biol.

[104] Song N, Xu H, Wu S, Luo S, Xu J, Zhao Q, Wang R, Jiang X (2023). Synergistic activation of AMPK by AdipoR1/2 agonist and inhibitor of EDPs-EBP interaction recover NAFLD through enhancing mitochondrial function in mice. Acta Pharm Sin B.

[105] Ren K, Su H, Lv LJ, Yi LT, Gong X, Dang LS, Zhang RF, Li MH (2019). Effects of four compounds from *Gentianella acuta* (Michx.) hulten on hydrogen peroxide-induced injury in H9c2 cells. Biomed Res Int.

[106] Li J, Wu J, Chen Q, Yu H, Liu M, Wang Y, Zhang Y, Wang T (2025). 7′-Hydroxyl substituted xanthones from *Gentianella acuta* revert hepatic steatosis in obese diabetic mice through preserving mitochondrial homeostasis. Biochem Pharmacol.

[107] Li W, Li Y, Siraj S, Jin H, Fan Y, Yang X, Huang X, Wang X, Wang J, Liu L (2019). FUN14 Domain-containing 1-mediated mitophagy suppresses hepatocarcinogenesis by inhibition of inflammasome activation in mice. Hepatology.

[108] Ma Z, Chen W, Liu Y, Yu L, Mao X, Guo X, Jiang F, Guo Q, Lin N, Zhang Y (2024). Artesunate Sensitizes human hepatocellular carcinoma to sorafenib via exacerbating AFAP1L2-SRC-FUNDC1 axis-dependent mitophagy. Autophagy.

[109] Ma Z, Zhang D, Sun J, Zhang Q, Qiao Y, Zhu Y, Niu J, Ren Q, Zhou L, Wen A, Wang J (2022). Formononetin inhibits hepatic I/R-induced injury through regulating PHB2/PINK1/Parkin pathway. Oxid Med Cell Longev.

[110] Kalavalapalli S, Bril F, Koelmel JP, Abdo K, Guingab J, Andrews P, Li WY, Jose D, Yost RA, Frye RF (2018). Pioglitazone improves hepatic mitochondrial function in a mouse model of nonalcoholic steatohepatitis. Am J Physiol Endocrinol Metab.

[111] Yang W, Zhao J, Zhao Y, Li W, Zhao L, Ren Y, Ou R, Xu Y (2020). Hsa_circ_0048179 attenuates free fatty acid-induced steatosis via hsa_circ_0048179/miR-188-3p/GPX4 signaling. Aging (Albany NY).

[112] Chen M, Huang F, Chen B, Kang J, Yao Y, Liua M, Li Y, Li Y, Zhou T, Peng D (2023). A classical herbal formula alleviates high-fat diet induced nonalcoholic steatohepatitis (NASH) via targeting mitophagy to rehabilitate dysfunctional mitochondria, validated by UPLC-HRMS identification combined with in vivo experiment. Biomed Pharmacother.

[113] Deng J, Long J, Yang Y, Yang F, Wei Y (2024). Gentiana decoction inhibits liver fibrosis and the activation of hepatic stellate cells via upregulating the expression of Parkin. Fitoterapia.

[114] Chen Y, Chen HN, Wang K, Zhang L, Huang Z, Liu J, Zhang Z, Luo M, Lei Y, Peng Y (2019). Ketoconazole exacerbates mitophagy to induce apoptosis by downregulating cyclooxygenase-2 in hepatocellular carcinoma. J Hepatol.

[115] Bano A, Chaker L, Plompen EP, Hofman A, Dehghan A, Franco OH, Janssen HL, Darwish Murad S, Peeters RP (2016). Thyroid function and the risk of nonalcoholic fatty liver disease: The rotterdam study. J Clin Endocrinol Metab.

[116] Bruinstroop E, Dalan R, Cao Y, Bee YM, Chandran K, Cho LW, Soh SB, Teo EK, Toh SA, Leow MKS (2018). Low-dose levothyroxine reduces intrahepatic lipid content in patients with type 2 diabetes mellitus and NAFLD. J Clin Endocrinol Metab.

[117] Zhou J, Sinha RA, Yen PM (2021). The roles of autophagy and thyroid hormone in the pathogenesis and treatment of NAFLD. Hepatoma Res.

[118] Harrison SA, Bashir MR, Guy CD, Zhou R, Moylan CA, Frias JP, Alkhouri N, Bansal MB, Baum S, Neuschwander-Tetri BA (2019). Resmetirom (MGL-3196) for the treatment of non-alcoholic steatohepatitis: A multicentre, randomised, double-blind, placebo-controlled, phase 2 trial. Lancet.

[119] Keam SJ (2024). Resmetirom: First approval. Drugs.

[120] Lee JH, Seo KH, Yang JH, Cho SS, Kim NY, Kim JH, Kim KM, Ki SH (2024). CCCP induces hepatic stellate cell activation and liver fibrogenesis via mitochondrial and lysosomal dysfunction. Free Radic Biol Med.

[121] Wei R, Cao J, Yao S (2018). Matrine promotes liver cancer cell apoptosis by inhibiting mitophagy and PINK1/Parkin pathways. Cell Stress Chaperones.

[122] Wu H, Wang T, Liu Y, Li X, Xu S, Wu C, Zou H, Cao M, Jin G, Lang J (2020). Mitophagy promotes sorafenib resistance through hypoxia-inducible ATAD3A dependent axis. J Exp Clin Canc Res.

[123] Yu SX, Liang ZM, Wu QB, Shou L, Huang XX, Zhu QR, Xie H, Mei RY, Zhang RN, Zhai XY (2022). A novel diagnostic and therapeutic strategy for cancer patients by integrating Chinese medicine syndrome differentiation and precision medicine. Chin J Integr Med.

